# Single-Molecule LATE-PCR Analysis of Human Mitochondrial Genomic Sequence Variations

**DOI:** 10.1371/journal.pone.0005636

**Published:** 2009-05-20

**Authors:** Adam Osborne, Arthur H. Reis, Loren Bach, Lawrence J. Wangh

**Affiliations:** Department of Biology, Brandeis University, Waltham, Massachusetts, United States of America; Ecole Normale Supérieure de Lyon, France

## Abstract

It is thought that changes in mitochondrial DNA are associated with many degenerative diseases, including Alzheimer's and diabetes. Much of the evidence, however, depends on correlating disease states with changing levels of heteroplasmy within populations of mitochondrial genomes, rather than individual mitochondrial genomes. Thus these measurements are likely to either overestimate the extent of heteroplasmy due to technical artifacts, or underestimate the actual level of heteroplasmy because only the most abundant changes are observable. In contrast, Single Molecule (SM) LATE-PCR analysis achieves efficient amplification of single-stranded amplicons from single target molecules. The product molecules, in turn, can be accurately sequenced using a convenient Dilute-‘N’-Go protocol, as shown here. Using these novel technologies we have rigorously analyzed levels of mitochondrial genome heteroplasmy found in single hair shafts of healthy adult individuals. Two of the single molecule sequences (7% of the samples) were found to contain mutations. Most of the mtDNA sequence changes, however, were due to the presence of laboratory contaminants. Amplification and sequencing errors did not result in mis-identification of mutations. We conclude that SM-LATE-PCR in combination with Dilute-‘N’-Go Sequencing are convenient technologies for detecting infrequent mutations in mitochondrial genomes, provided great care is taken to control and document contamination. We plan to use these technologies in the future to look for age, drug, and disease related mitochondrial genome changes in model systems and clinical samples.

## Introduction

Mitochondria are the primary energy source of most eukaryotic cells. Each mitochondrion possesses multiple copies of mitochondrial DNA (mtDNA). Human mitochondrial DNA is a 16 kb circle that encodes for 13 genes electron transport chain proteins, 22 tRNAs, and two rRNAs. The mitochondrial genome also includes a control region that contains the displacement loop (D-Loop), within which DNA replication is initiated and gene transcription is regulated. By convention one particular sequence, known as the revised Cambridge Reference Sequence (rCRS), or the Anderson sequence, serves as a reference sequence [1], [2]. Variations from this reference sequence at specific loci within the HVI and HVII regions of the D-Loop define each person's haplotype. Haplotypes are used for forensic identification, and sequence changes with respect to the haplotype can, at least theoretically, be used to analyze mitochondrial diseases. Recently, mutations within the D-Loop of mtDNA have been linked to specific diseases [3] including diabetes [4], Alzheimer's [5], and cancer [6]. There are multiple mechanisms that could cause disease: a single point mutation that is clonally expanded throughout the cell, or a build up of random mutations (mutational load) that, having reached a threshold, causes the onset of disease. Amplification of bulk samples will only show the most abundant mutations which may or may not play a significant role in disease development under mutational load conditions. In order to observe the increase in mutations over time it is necessary to amplify single mtDNA molecules. Studies using such high resolution analysis are pivotal to understanding the relationship of heteroplasmy to disease.

Published evidence for mtDNA heteroplasmy is controversial for several technical reasons [7], [8]. First, sequencing errors and contamination can lead to overestimating the extent of heteroplasmy in a sample. Recently it has been shown by Yao *et al.* that even single cell analysis of mtDNA is problematic due to contamination within the laboratory space [9]. Even when adequate controls for these artifacts are in place, most mtDNA studies have involved PCR amplification starting with populations of molecules followed by cloning or direct cloning of individual mtDNA genomes prior to amplification and analysis [10]–[20]. The use of populations of mtDNA detects only high frequency variants within the population, while cloning makes it difficult to distinguish between mutations (hereafter, true mutations) in the starting genomes and those introduced during the construction and selection of clones.

Both forms of cloning, PCR amplified or direct, have problems analyzing single molecules. With direct cloning it is impossible to distinguish between true mutations and mutations that occurred during replication of the plasmid. With PCR amplification first, any errors that take place during the PCR will be included in the pool of cloned molecules, thereby making it impossible to distinguish between true mutations and PCR errors.

Random mutation capture (RMC) [21], [22] helps reduce the number of molecules in a population and avoids cloning by using restriction digestion. Wild type molecules at the enzyme-specific sequence are digested, while mutant molecules are not. These remaining molecules are then amplified. This technique has several draw backs. The first is that RMC actually works best at a population level and therefore suffers from not being able to measure the breadth of mutations that may make up mutational load at the single copy level. Second, most mutations of interest occur outside the sequence cleaved by a single restriction enzyme. This means that to survey widely distributed mutations multiple restriction enzymes would have to be used in a manner that does not interfere with subsequent PCR amplification of the resulting fragments.

Direct amplification of single molecules followed by sequencing, allows each strand of mtDNA to be individually analyzed, thereby illuminating possible heteroplasmic differences between genomes [23], [24]. This approach makes it possible to detect low frequency genetic changes at virtually any base pair. SM-LATE-PCR takes advantage of the fact that LATE-PCR allows for efficient and rapid generation of single stranded DNA [25], [26]. Dilute-‘N’-Go sequencing, in turn, allows for convenient dideoxy-sequencing of the accumulated single-stranded DNA, as well as the template strand from which it was generated [27].

Using LATE-PCR and Dilute-‘N’-Go sequencing, we have shown that it is possible to detect mutations in the HVI region of mtDNA at a single molecule level in hair samples of a 60 year old male. Low numbers of true mutations were found based on a conservative interpretation that any sequence that does not maintain the same baseline sequence (rCRS or rCRS with changes) as the original amplified bulk sample (this predominant sequence is denoted as HV1_∞_) is likely to be an artifact due to sample contamination. Indeed, in initial experiments high numbers of contaminates were found, similar to recent studies [9]. This observation led to a series of experiments designed to establish: i) where the contamination was coming from, and ii) if SM-LATE-PCR or Dilute-‘N’-Go sequencing were a source of artifacts. Our data demonstrate that neither of these new techniques introduces detectable changes in DNA sequences and that the contamination observed came from within our laboratory. These SM-LATE-PCR experiments establish both the technology and the baselines needed to explore heteroplamy in the future under rigorously controlled laboratory conditions.

## Results

### Analysis of Single mtDNA Molecules from Hair Shafts

This study was initiated using a 5 mm portion of a hair shaft from a 60 year old male. The strand was first cleaned carefully and was then lysed to avoid contamination (see methods). No hair root was present, thereby ruling out the possibility of contamination with nuclear genomic DNA, which is known to contain pseudogenes for mitochondrial genes [28].

**Figure 1:**
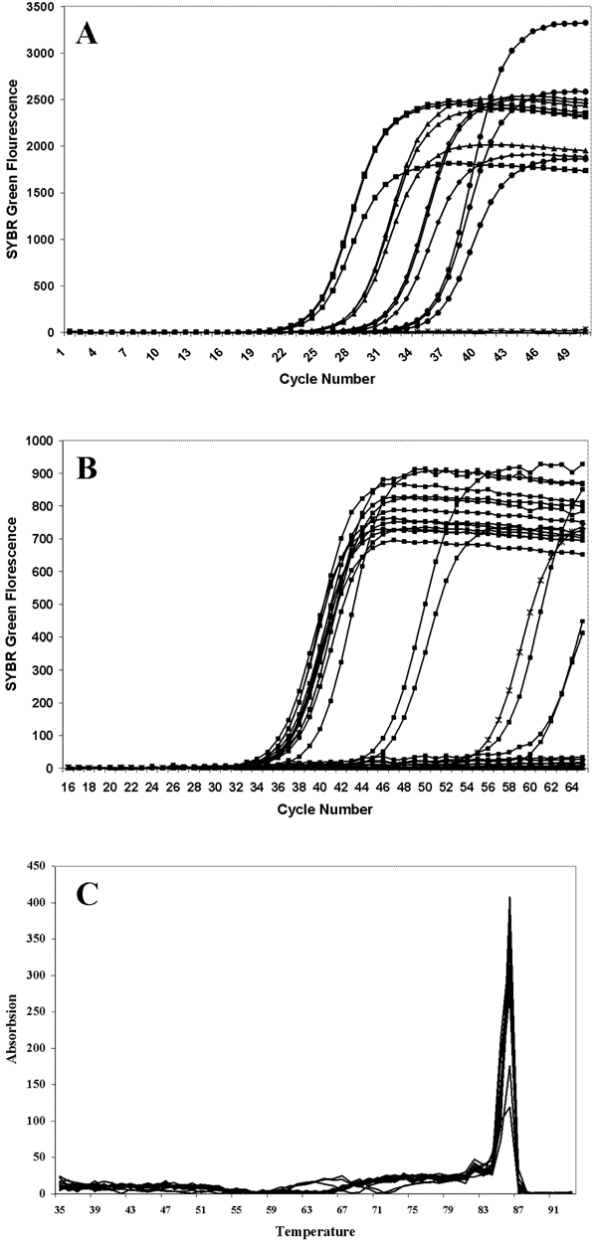
Amplification plots of (a) dilution series and (b) single molecules using blood lymphocyte mtDNA. (a) (*) equal NTC, (▪) 10,000 copies, (♦) 1,000, (▴) 100, and (•) 10 copies. (b) Single copy amplification has scattered Ct values. (▪) equal single copy mtDNA, (*) equal NTCs. (c) SYBR Green melts of HV1 samples. The predicted melt of the amplicon is 86.5. The samples do melt with a sharp peak at that temperature showing that the sample has been amplified not only correctly but also cleanly.

In order to achieve SM-LATE-PCR, we diluted the population of mtDNA genomes in ten-fold steps (Figure 1a). The observed ΔC_t_ value at each step of the dilution series was about 3, confirming that the LATE-PCR assay for amplification of HVI was efficient [25]. Replicate samples diluted below 10 molecules per assay have scattered C_T_ values because the presence of a genome per tube becomes stochastic (Figure 1b). The double-stranded DNA melted at the temperature expected for our HVI amplicon, 86.5°C (Figure 1c). The predominant HV1 sequence of the undiluted population of genomes, hereafter designed HVI_∞_, differed from the rCRS in four locations: 16136C, 16224C, 16311C, and 16320T (Tables 1 and 2).

**Table 1:** List of results for hair shaft mitochondria experiments.

Experiment (Number of Wells)	Total Number of Wells That did not Amplify	Number of Samples That Have the HV1_∞_ Sequence	Sample Name	Number of Changes from HV1_∞_
Hair Shaft (96)	68	18	M1	–
			M2	8
			M3	8
			M4	8
			M5	8
			M6a	8
			M6b	–
			M7	10
			M8	1
			M9	2
			NTC	3
NTC 1 (96)	91	NA	1	8
			2	8
			3	6
			4	6
			5	5
NTC 2 (96)	93	NA	1	5
			2	8
			3	5
NTC 3 (96)	94	NA	1	8
			2	8

Samples with an a or b following mean that they were two sequences in the same tube. (–) There were no mutations.

**Table 2:** Table of sequences from the Hair amplifications.

Sample	Sequence
Experimenter	16224C–16304C–16311C
Hair	16136C–16224C–16311C–16320T
M2, 3, 4, 5	16129A–16136T–16192T–16223T–16224T–16297C–16311T–16320C
M6	16129A/G–16136T/C–16192T/C–16223T/C–16224T/C–16297C/T–16311T/C–16320C/T
M7	16111T–16129A–16136T–16223T–16224T–16290T–16311T–16319A–16320C–16362C
M8	16187T
M9	16030T–16125T

Changes for the Hair and Experimenter are based off of the Anderson sequence. The changes for the hair mutations are based off of the Hair sequence, which makes up the HV1_∞_ sequence. Heteroplasmy is indicated by a/.

SM-LATE-PCR was carried out by diluting the sample to slightly less than the theoretical value of one molecule per well. Accordingly, we recovered 28 positive signals out of 90 samples when the sample was diluted to the single molecule level and amplified by SM-LATE-PCR (Table 1). Two of the resulting 28 experimental sequences were clear contaminants: (M1) was the Anderson Sequence and (M7) was a sequence with which we were familiar from earlier experiments. M7 differed from the HVI_∞_ sequence at the following sites: 16111T, 16129A, 16223T, 16290T, 16319A, and 16362C (Table 2). Of the remaining 26 sequences 18 were identical to the HVI_∞_ sequence. Of the remaining 8 sequences, 4 (M2–M5) contained a sequence of 16129A, 16136T, 16192T, 16223T, 16224T, 16297C, and 16320C rather than HVI_∞_ sequence (Table 2). The M2–M5 results, as well as the one NTC that amplified, were considered contamination because they did not contain the same changes relative to the rCRS as those that defined the HVI_∞_ sequence. Sample M6a/M6b contained equal amounts of both the HVI_∞_ sequence and the sequence seen in samples M2–M5.

The final two samples in this set (M8 and M9) had the HVI_∞_ sequence but also had one (M8) or two (M9) additional base changes. Even though the base changes in the M9 sequence have not been previously observed, they fit the definition of true mutations, that is changes that do not alter the baseline HVI_∞_ sequence. Although the sample size is very small, the rate of change, two out of twenty-eight samples (7%), is consistent with the average mutation rate reported by others [28]. Nevertheless, the results obtained with the hair shaft sample were vexing because they demonstrated that >8% (8 out of 90 total sample reactions) of the amplified reactions were likely due to contaminants from several sources, despite our efforts to avoid this problem. In response we took a closer look at the possible sources of contamination in these experiments.

### Analysis of Single mtDNA No-Template Controls

In order to analyze the contamination problem in depth we began by amplifying a large number of non-template controls to determine whether our reagents or the laboratory environment was the source of the problem. Accordingly, 288 control samples were analyzed using the SM-LATE-PCR protocol. These controls were run on two different PCR machines with fresh reagents each time. Among the first 96 reactions, NTC-1, five wells generated a signal. Two of these sequences had eight additional changes from the canonical rCRS sequence (Table 1 and 3). The other three positive reactions, samples 3, 4, and 5, had 6, 7, and 5 changes respectively (Tables 1 and 3). None of the five NTC sequences obtained in this experiment match the dominant HVI_∞_ sequence of the hair sample.

**Table 3:** Table of sequences from the NTC amplifications.

NTC-1-1, -2	16048A–16051G–16092C–16209C–16239T–16278T–16352C–16353T
NTC-1-3	16126C–16158G–16163G–16186T–16189C–16294T
NTC-1-4	16167T–16192T–16223T–16298C–16311C–16325C–16327T
NTC-1-5	16126C–16293G–16294T–16296T–16304C
NTC-2-1	16129A–16192T–16223T–16297C–16303C
NTC-2-2	16048A–16051G–16092C–16209C–16239T–16278T–16352C–16353T
NTC-2-3	16122G–16126C–16294T–16296T–16304C
NTC-3-1, -2	16048A–16051G–16092C–16209C–16239T–16278T–16352C–16353T

NTC-1, NTC-2, and NTC-3 are the first, second, and third NTC amplification runs. All changes are based off of the Anderson sequence.

Among the 96 NTC-2 reactions, three generated signals. Two of these three had sequences observed in NTC-1. The third sample had five changes (Table 3). Among the 96 NTC-3 reactions, 2 generated signals and both matched the NTC-1-1, NTC-1-2, and NTC-2-2 sequences seen in the first two NTC plates. Indeed, these sequences are identical to those observed in earlier mitochondrial work in our laboratory on blood lymphocytes, even in the number and location of sequence changes.

The results from the NTC reactions indicate that some of the contamination we observed came from amplicons released during the earlier work on mitochondrial genomes. Having established this pattern we can justifiably discount those sequences within the set from hair shaft as cases of contamination rather than as true mutations. This line of reasoning also supports our general conclusion that samples that show several sequence changes from the predominant sequence of that sample are likely to be contaminants of unknown origin. None of the contaminants picked up in these NTC experiments included the dominant sequence of the hair shaft experiments. We regard this as a positive indication that our current laboratory procedures are significant improvements over our initial ones.

### PCR and Sequencing Errors

Before drawing final conclusions from the above data, it was important to demonstrate both the fidelity of the SM-LATE-PCR amplification and the accuracy of Dilute-‘N’-Go dideoxy-sequencing by understanding the distribution of amplicon sequences in an amplified single molecule sample. Both of these technologies were validated by mitochondrial DNA from samples of hair shaft. Accordingly, original hair shaft sample M2 LATE-PCR generated amplicons were diluted to a single amplicon per microliter. LATE-PCR amplification was repeated on 90 samples and analyzed for sequence similarity to the original M2 amplicon sample.

Sample M2 was selected to determine if SM-LATE-PCR and Dilute-‘N’-Go sequencing generated errors because it was one of four amplified hair samples that had the same sequence. While the M2–M5 samples were considered contaminants, it is difficult to classify them as sequencing or amplification errors because the same eight base changes were very unlikely to have arisen independently in each of four reactions. Thirty-four of the diluted M2 amplicons generated SM-LATE-PCR products. Twenty-four of these samples had the same sequences, thereby defining HVI_∞M2_. The other ten sequences each had individual changes while retaining the HVI_∞M2_ sequence (Table 4). *A priori*, these additional changes could either be the result of errors during Dilute-‘N’-Go sequencing or could be the result of errors during the first round of SM-LATE-PCR amplification.

**Table 4:** Table of sequences from the re-amplification of the M2 sample.

M2	16129A–16136T–16192T–16223T–16224T–16297C–16311T–16320C
M2-1	16048C
M2-2	16229A/T–16258G/A–16260T/C
M2-3	16100G
M2-4	16171G
M2-5	16175G
M2-6	16239T/C
M2-7	16137G–16180G
M2-8	16051G
M2-9	16023A
M2-10	16297T/C

All changes are based off of the M2 sequences. Heteroplasmy is indicated by a/.

**Figure 2:**
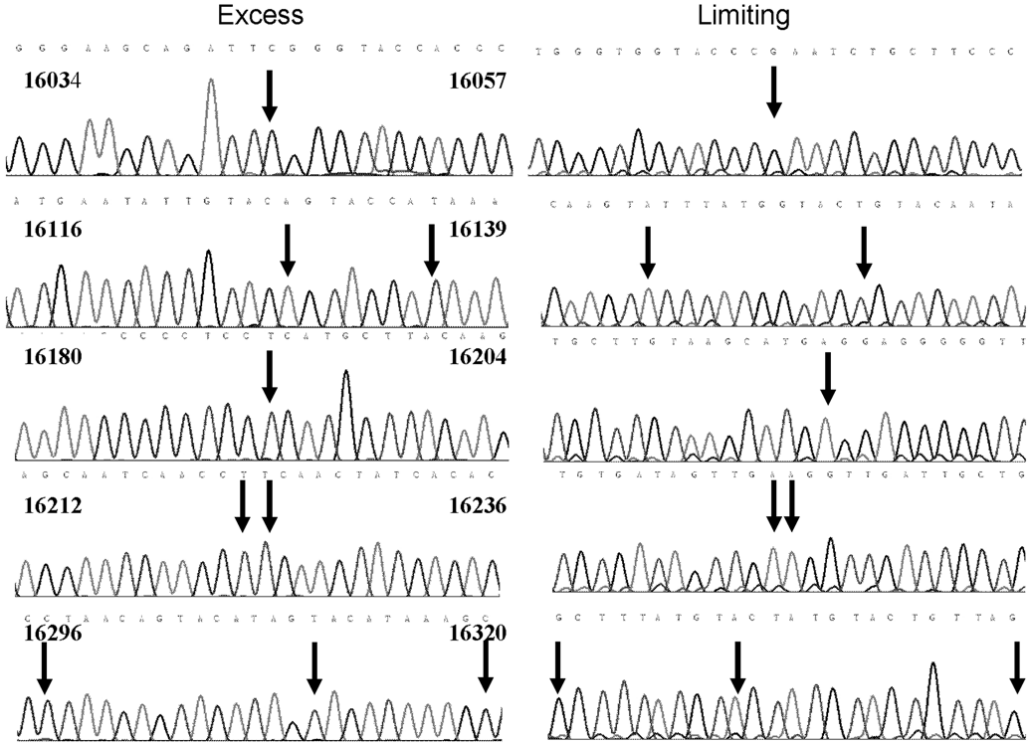
Sequencing chromatograms of Sample M2-1. Both excess and limiting primers have been used to sequence the amplicon. What are shown are the complementary sequences. Sequence ranges are given at the top of each section. Mutations have been indicated with black arrows. The background seen when using the limiting strand is the product produced with what excess primer is left over after dilution.

Errors due to Dilute-‘N’-Go sequencing of the Excess Primer strand were ruled out by sequencing the complementary Limiting Primer strand that is present in each sample. In every case the Limiting Primer strand was fully complementary to its Excess Primer strand (Figure 2). Therefore the sequence changes of the ten HVI_∞M2_ were present in the amplicons themselves prior to sequencing, were not due to sequencing errors, and would never have been detected in bulk mtDNA amplifications (see below).

Each of the sequence changes observed in these 10 errant HVI_∞M2_ samples were non-heteroplasmic at each altered position. This means that they could not have arisen early in the process of SM-LATE-PCR amplification for the following reason. When amplification is initiated from a single molecule any base change occurring during the first cycle of duplication results in a 50∶50 mixture of sequences and any base change during the second cycle of duplication results in a 75∶25 mixture of sequences. Mixtures at both of these levels are distinguishable from non-heteroplasmic during sequencing. Amplification errors that arise during later cycles do not leave a detectable trace by sequence analysis because dideoxy-sequencing uses populations of template molecules.

We conclude that the 10 errant HVI_∞M2_ samples were present together with the 24 unchanged HVI_∞M2_ molecule in the 34 template molecules recovered from sample M2. We further conclude that these 10 errant HVI_∞M2_ molecules were produced between the 3^rd^ and final round of double-stranded duplication in the SM-LATE-PCR assay used to generate sample M2. As discussed below the error rate of SM-LATE-PCR detected here is consistent with the inherent error rate of symmetric PCR.

## Discussion

Our long range goal is to accurately measure the levels of heteroplasmy in mitochondrial genomes in relation to several degenerative diseases. As a first step toward this goal, we analyzed single molecules of mtDNA from a hair shaft in order to validate SM-LATE-PCR. During this experiment we detected both true mutations and mtDNA contamination. These findings led us to carry out experiments aimed at revealing the source of contamination and this analysis, in turn, allowed us to conclude that the contamination problem largely arose from early laboratory procedures. But, like Yao *et al.* [9], we also detected contamination from unknown sources. Our final series of experiments proved to be the most important for future research, because it demonstrated that Dilute-‘N’-Go dideoxy-sequencing is reliable and that the error rate inherent to SM-LATE-PCR is consistent with that observed in symmetric PCR.

### Single Molecule LATE-PCR Amplifications

We have chosen to define true mutations based on a conservative interpretation of the data. True mutations are defined as a change in a DNA sequence at a location other than the defining HVI_∞_ sequence characteristic of that sample. Thus, two of twenty-eight hair shaft sequences were scored as true mutations. This low rate of true mutation, 7%, is similar to the mitochondrial HVI mutation rate reported using other methods [29].

Our experiment also revealed the presence of contaminating sequences in each sample set. But all of the contaminating sequences could be traced to early experimental work in our laboratory. While it is probably impossible to rid our laboratory of these contaminants, we can justifiably discount their presence in the future.

We conclude that SM-LATE-PCR is an extremely sensitive method, and experience has taught us that amplification of non-template controls prior to analysis of experimental samples is critical in order to calibrate contaminants present in the laboratory space and in reagents. Indeed, NTC amplifications may have to be done repeatedly during experiments that are extended over a long time. This requirement is particularly stringent when human mitochondria are being analyzed.

### Heteroplasmy Is Not Due to Sequencing Errors

Single molecules of mtDNA were amplified and sequenced bi-directionally using Dilute-‘N’-Go dideoxy-sequencing. This showed that sequencing errors do not occur using this novel protocol. If such errors had taken place, the complementary sequencing runs would have been non-complementary at the site in question. When the Excess Primer Strand is used as the sequencing template, there is a very low level of background signal in the chromatogram. This background may be due to the Limiting primer-strand, because some Excess Primer is still present even after 1∶20 dilution, and can still be extended during cycle sequencing. But, this low background never results in a false base call or a sequence ambiguity. No background is seen when the Limiting Primer Strand is sequenced with the residual Excess primer, because there is no Limiting primer left to prime the Excess Primer Strand.

### Heteroplasmy Is Not Due to Amplification Errors

Sequence differences among replicate SM-LATE-PCR assays are not due to amplification errors for the following reasons. LATE-PCR begins with symmetric PCR and then switches to repeated cycles of linear amplification in which the Limiting Primer-Strand serves as the template but does not itself replicate further. Thus, only amplification errors that arise very early during the first phase of double-stranded DNA amplification could measurably impact the composition of the population of single-stranded amplicons that serve as templates for post-amplification sequencing. In fact, when amplification begins with a single molecule, an amplification that changes sequence would have to occur during the very first or second cycle of amplification to subsequently be detected via dideoxy-sequencing. Dideoxy-sequencing, including Dilute-‘N’-Go dideoxy-sequencing, generates and linearly amplifies dideoxy-partial sequences from a population of template molecules. For instance, if the initial template is comprised of a 50∶50 mixture of two sequences upon sequencing the heteroplasmy is seen in the sequence results (M6 in Table 1). However, the sensitivity of dideoxy-sequencing for detection of heteroplasmy quickly falls off, i.e. 75∶25 mixture of two sequences are detectable, but 88.5∶12.5 mixture are usually not. It follows from these observations that pure sequence difference among different samples generated by SM-LATE-PCR must have been present in the initial population of molecules. This idea is further supported by earlier work on single molecule PCR [23].

### Symmetric PCR is Known to Generate Sequence Errors

Lin *et al.* calculated the rate of PCR-induced mutation to be 1.18×10^−4^ errors/bp [30]. While that study used symmetric PCR rather than LATE-PCR, the calculations were based on the error insertion rate of a high-fidelity DNA polymerase, the same enzyme as was used for amplifications discussed in this study. Thus, it can be reasonably assumed that LATE-PCR will exhibit a similar rate of error during the initial symmetric phase of amplification. It is extremely unlikely that any of the sequence changes that we scored as a “true” mutation was due to a PCR amplification error. Were this to be the case, they would have to occur during either the first or second round of amplification for the reasons outlined above.

### Nevertheless SM-LATE-PCR Does Generate Sequence Changes During Amplification

Although none of the heteroplasmy we observed after one round of SM-LATE-PCR is explained as an artifact during early amplification, this does not rule out the possibility that sequence changes do occur at some point during SM-LATE-PCR. In order to rigorously test this possibility, one of four identical samples generated by a first round of SM-LATE was diluted to the single amplicon level. Replicates of these secondary single amplicons were again subjected to SM-LATE-PCR and Dilute-‘N’-Go sequencing. Our results show that, this time, 10 of 34 samples were errant version of HVI_∞M2_ with 8 versions containing single base changes. Using the above error rate from Lin *et al.*, the expected number of sequences with errors would be 6. We conclude that these 10 errant HVI_∞M2_ samples reflect the presence of at least 28.5% (10 out of 34) sequence variants generated by the first SM-LATE-PCR amplification experiment on hair.

It follows from the above analysis that the first SM-LATE-PCR amplification from an initial population of diluted mitochondrial genomes provides an accurate picture of the sequence heteroplasmy within that population of genomes. However, the resulting populations of amplicons generated by SM-LATE-PCR include hidden sequence changes that arise during this first amplification. It is important to emphasize, however, that none of the “true mutations” described herein, nor any of the “contaminants” described herein reflects these hidden sequence changes because all such conclusions were drawn from first round amplification of single mitochondrial genomes.

#### Conclusions and Look to the Future

Applications for mitochondrial research at the single genome level are numerous and range from exploration of the mutation level present in single cells and mitochondrion, to examination of the potential mechanisms of mtDNA related disease. In-depth analysis requires the use of a reliable method of single mtDNA amplification and sequencing with the minimum chance of contamination. We have shown here that SM-LATE-PCR and Dilute-‘N’-Go Sequencing is such a method, that is not only useful but also does not generate additional errors or suffer from contamination to any greater extent than previously published reports [9], [30]. Assays of heteroplasmy among different tissues, between similar tissues at different stages of life, or among maternally related individuals may potentially lead to rigorously established rates of heteroplasmy that make it possible to track disease relationships between groups of related and unrelated people for the first time. LATE-PCR is a newly emerging technique that has accounted for the problems inherent in conventional PCR. We have proven here that LATE-PCR can reliably amplify single molecules of mtDNA. This work also sets the bounds and controls needed for future SM-LATE-PCR work. With strict controls in place the combination of SM-LATE-PCR and Dilute-‘N’-Go sequencing will likely emerge as a powerful new analytical technique for single molecule analysis of mitochondrial DNA.

## Materials and Methods

### MtDNA extraction

#### Cell sampling techniques

Mitochondrial DNA from hair shaft samples were extracted and prepared after approval from the Brandeis University Human Subjects Institute Review Board and the Brandeis University Biosafety Committee. For the hair shaft sample, one hair, approximately 15 mm in length, with the root removed, was cut into three sections. Each section was washed in 5% Liqui-Nox solution followed by two washes in water and ethanol. No further separation procedures were required for hair shaft samples prior to cell lysis.

#### Cell lysis prior to amplification

Cell lysis for hair shaft was achieved using a mixture of mixture of 96 µL of Quantilyse and 4 µL of dithiothreitol (DTT) (Sigma Gynosys™). Quantilyse is a proteinase K containing lysis buffer prepared in the Wangh lab [31]. Extracted cells were suspended in the 100 µL lysis mixture in a 250 µL clear Applied BioSystems (ABI) reaction tube. The 250 µL tube was heated to 50°C for 2 hours then at 95°C for 15 minutes to ensure that all cellular components were entirely broken down.

### LATE-PCR protocols: reactants and reaction conditions

All PCR preparation was conducted in an amplicon free hood in a clean room. All targets are kept separate from PCR reagents at all times. PCR machines are kept in a separate room, from the laboratory space, and as far away from the clean room as possible. PCR amplicons and products are never handled in the laboratory space.

#### HVI assay primers and probe

The amplicon produced in amplifications of the HVI region was 549 bases in length between bases 16458 and 15910 based on the revised Cambridge Reference Sequence (rCRS). The excess primer (5′- CACCAGTCTTGTAAACCGGAGATGAA - 3′ target 15910–15935) had a concentration-adjusted T_m_ of 62°C and was present in each reaction at a concentration of 1 µM. The limiting primer (5′ - GCCCGGAGCGAGGAGAGTAGCACTCTTG - 3′ target 16340–16458) had a concentration adjusted T_m_ of 68°C and was present in a concentration of 0.05 µM.

#### Reactants and reaction conditions

All amplifications were carried out in the ABI prism 7700, NTC samples were also carried out in the BioRad IQ5, sequence detection system at a volume of 25 µL. The thermal profile was the same for all amplification reactions and melts, as this profile was calibrated to primer, and amplicon T_m_ values. The reaction mixture was initially heated to 95°C for 3 minutes, then 65 cycles of 95°C for 15 seconds, 64°C for 10 seconds, and 72°C for 45 seconds. Accumulation of dsDNA accumulation was monitored at 72°C using SYBR® Green intercalating dye at a concentration of 1× (FMC Bioproducts Rockland, ME). DNA melts assess sample purity by monitoring the SYBR® Green fluorescence signal as the temperature gradually increased from 35°C to 95°C in 60 15 second cycles where the temperature increased by 1 degree each cycle.

#### Reactants

Each 25 µL LATE-PCR reaction mixture consisted of 10× PCR buffer at a 1× concentration (Invitrogen), 50 mM MgCl_2_ at 3 mM (Invitrogen), 10 mM dNTP's at 250 µM (Promega), 10× SYBR® Green (FMC Bioproducts) at 1× concentration, 10 µM limiting primer at 0.05 µM or 50 nM (Operon), 100 µM excess primer at 1 µM, 10 uM 045 PrimeSafe at 0.5 µM or 500 nM (Operon), 1.25 units of Pt Taq (Invitrogen), de-ionized and DNA-free water (Invitrogen), and 1 µL of target mtDNA suspended in Tris HCl buffer pH 8.3 (Sigma).

### Dilute-‘N’-Go sequencing Preparation and Conditions

#### Preparation

The amount of ssDNA amplicon generated during the linear phase of LATE-PCR is generally 100 to 200 times more than is needed for dideoxy-sequencing reactions [32]. Amplified samples were handled and diluted in a separate room well away from the main laboratory space (in this case in a room on a different floor that is vented to the outside), to a standard concentration of approximately 25 fm/µL using Tris HCl pH 8.3. This diluted product was then used in aliquots of 4 or 8 µL for a total of 100 fm or 200 fm per sequencing tube.

Each sequencing sample contained 20 µL of de-ionized, DNA-free water (Invitogen), and 4 µL of the diluted ssDNA product in Tris HCl Buffer pH 8.0 (Sigma). In heteroplasmy experiments where pipetting very small (1 µL) amounts of mtDNA had the potential to reduce the accuracy of mixture ratios samples were diluted down to 6.25 fM/µL rather than 25 fM/µL. While the total amount of mtDNA in the prepared tube remained the same (100 fM), the pre-sequencing mixture contained 8 µL of water, 1 µL of limiting primer and 16 µL of diluted ssDNA amplicon mixture.

#### Sequencing

Dideoxy-sequencing was conducted in the Brandeis Biochemistry Core Facility on a CEQ:2000XL model 373 DNA Sequencing System (Applied Biosystems) [33]. Sequences for the validation experiments were diluted to 50 fM [27] and sent to Genewiz, Inc. (New Jersey).

#### Sequence analysis

Sequence comparison was conducted through the CLUSTAL program available online at http://www.ebi.ac.uk/clustalw. The program aligns and scores (percent sequence similarity) sequences facilitating base by base comparison of replicate samples as well as comparison of sequence samples against an established standard. The conventional sequence used as a basis of comparison for the determination of mtDNA haplotype is the revised Anderson sequence also known as the revised Cambridge Reference Sequence (rCRS) which is available at http://www.mitomap.org/. Polymorphic or heteroplasmic sites were examined using Chromas version 2.31, a chromatogram viewing program by Technylysium Pty. Ltd.
